# GL-V9 Promotes Autophagy-Mediated YAP1 Degradation and Activates Mitochondrial Apoptosis in PDAC Cells

**DOI:** 10.3390/ph17101352

**Published:** 2024-10-10

**Authors:** Hao Liu, Zhangxing Lin, Yongjian Guo, Yuxin Zhou, Wei Li

**Affiliations:** 1 School of Integrated Chinese and Western Medicine, Nanjing University of Chinese Medicine, 138 Xianlin Avenue, Nanjing 210023, China; 20210019@njucm.edu.cn; 2 State Key Laboratory of Natural Medicines, Jiangsu Key Laboratory of Carcinogenesis and Intervention, China Pharmaceutical University, 24 Tongjiaxiang, Nanjing 210009, China; 3320092055@stu.cpu.edu.cn (Z.L.); guoyj@cpu.edu.cn (Y.G.)

**Keywords:** PDAC, YAP1, autophagy, GL-V9, mitochondrial apoptosis

## Abstract

**Background:** Pancreatic ductal adenocarcinoma (PDAC) is among the most aggressive forms of pancreatic cancer with a poor prognosis. YAP1 expression is markedly elevated in PDAC, but how it works is not clear. GL-V9, a derivative of the natural compound wogonin, effectively fights a variety of tumors; however, its effect on PDAC has not yet been studied. **Methods:** TCGA database analysis, Western blots, immunofluorescence, and real-time PCR were used to evaluate GL-V9’s effect on YAP1 expression and mRNA levels. Immunofluorescence was used to examine the co-location of YAP1 with LAMP2 and p62. Co-immunoprecipitation was used to assess the binding of YAP1 to ubiquitin, p62, and TEAD1. A PDAC graft tumor model was used to test GL-V9’s pharmacological effects. Western blots and immunohistochemistry were used to measure apoptosis- and autophagy-related protein expression. **Results:** GL-V9 effectively promoted the degradation of YAP1, reduced YAP1 nuclear localization, and induced mitochondrial apoptosis in PDAC cells. YAP1 overexpression led to the upregulation of Bcl-2 and attenuated the caspase cascade induced by GL-V9. Furthermore, we demonstrated that GL-V9 induced autophagosome–lysosome fusion via the AKT/mTOR/TFEB pathway, leading to mitochondrial apoptosis in PDAC cells. In vivo studies also confirmed that GL-V9 exerts anti-tumor effects by suppressing YAP1 expression, while also activating autophagy and inducing mitochondrial apoptosis in BXPC-3-bearing BALB/c nude mice. **Conclusions:** Our findings underscore the importance of autophagy-mediated YAP1 degradation in PDAC, providing a novel molecular rationale (GL-V9) as a promising treatment for this disease.

## 1. Introduction

Pancreatic ductal adenocarcinoma (PDAC) is characterized by its heterogeneity and currently lacks a definitive cure [1,2]. However, the lack of biomarkers, along with early metastasis, high drug resistance, significant cytotoxicity, and radiotherapy insensitivity, in PDAC pose formidable challenges for therapeutic approaches, resulting in poor patient prognosis [3]. Among the various apoptotic signaling pathways, the mitochondrial apoptosis pathway plays a crucial role in PDAC cells. Mitochondrial apoptosis and ROS-induced oxidative stress are integral to pancreatic cancer pathophysiology. In PDAC, excessive ROS disrupts cellular homeostasis [4], triggering cascading events such as a reduction in mitochondrial membrane potential, damage to mitochondrial DNA, and inhibition of mitochondrial respiratory chain enzymes, ultimately leading to cell death or mitochondrial autophagy [5,6]. In PDAC, the imbalance of ROS levels can lead to cellular damage and affect mitochondrial function, potentially triggering autophagy. Autophagy, a cellular degradation process, can serve dual roles, either promoting cancer cell survival under stress conditions or leading to cell death [7]. Autophagy promotes the proliferation and survival of advanced pancreatic cancer by degrading and recycling organelles and proteins. Clinically, autophagy of both the tumor and host cells contribute tumor cell growth promotion [8]. Apoptosis and autophagy are primary cellular mechanisms that regulate pancreatic cancer progression and maintain intracellular homeostasis.

The PI3K/Akt/mTOR pathway is essential in the physiology and pathology of PDAC [9], and its excessive activation has been reported in nearly all solid tumors, including pancreatic cancer [10,11,12]. In PDAC, the abnormal activation of the PI3K/Akt/mTOR pathway inhibits autophagy initiation by promoting mTORC1 activation through AKT. Thus, targeting this pathway may reactivate autophagy [13]. The transcriptional co-activator YAP is critical for balancing autophagy and apoptosis. It is critical for the onset and advancement of various solid tumors, including pancreatic cancer [14,15]. Furthermore, increased autophagy facilitates the degradation of YAP. Activation of the Hippo pathway promotes the phosphorylation of YAP/TAZ by LATS1 and LATS2, causing their sequestration in the cytoplasm. Subsequent ubiquitination and degradation via the proteasome or autophagy pathways prevent their nuclear translocation and interaction with the transcription factor TEAD, thereby inhibiting downstream target gene expression [16]. YAP and TAZ promote pancreatic cancer progression, independent of *KRAS* mutations [17], and are implicated in metastatic progression. A robust stromal response influences tumor growth and immune evasion. Recent findings indicate that YAP and TAZ modulate pancreatic cancer pathogenesis by regulating pancreatic stellate cell states and recruiting tumor-associated macrophages and myeloid-derived suppressor cells [18]. Moreover, YAP and TAZ are implicated in chemotherapy resistance and poor prognosis in pancreatic cancer [19]. Considering YAP1’s role in cancer biology, it may offer novel insights for targeted therapies.

GL-V9, a synthetic derivative of wogonin, exhibits promising anticancer effects (such as breast cancer, liver cancer, colorectal cancer, and T-lymphoblastoma) by inducing apoptosis and autophagy, as shown in our previous studies [20,21,22,23,24]. Recently, in the development of anti-pancreatic cancer drugs, we discovered that GL-V9 also has a good therapeutic effect on PDAC, but its mechanism remains unclear. Here, we investigated the therapeutic effects of GL-V9 on PDAC and elucidated its specific anticancer mechanisms, providing strong evidence for its expanded therapeutic use. Our study demonstrated that GL-V9 enhances cellular autophagy via the AKT/mTOR/TFEB pathway, leading to the degradation of YAP1 and the subsequent initiation of apoptosis, thereby inhibiting PDAC growth. These findings provide theoretical evidence for developing GL-V9 as a potential therapeutic agent for PDAC.

## 2. Results

### 2.1. GL-V9 Exhibits Potent Anticancer Activity in PDAC Cells by Inducing Mitochondrial-Mediated Apoptosis

**Figure 1:**
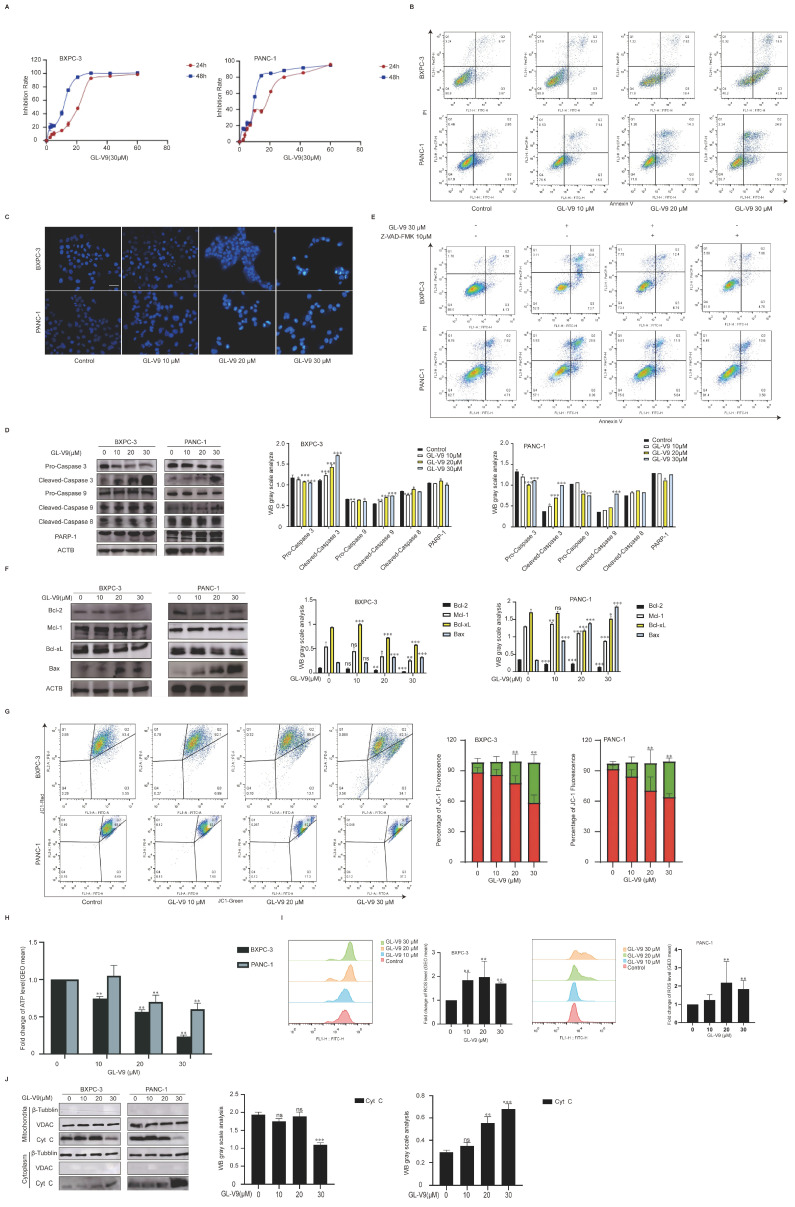
GL-V9 has potent anticancer activity in PDAC cells via inducing mitochondrial-mediated apoptosis. (**A**) MTT assay was used to examine the survival inhibition of pancreatic cancer cells (BXPC-3 and PANC-1) after treatment with different concentrations of GL-V9 for 24 h or 48 h; (**B**) flow cytometry was used to evaluate Annexin V-FITC/PI double staining following the treatment of BXPC-3 and PANC-1 cells with 0, 10, 20, and 30 μM GL-V9 for 24 h; (**C**) DAPI staining analysis of cell nuclei after treatment of BXPC-3 and PANC-1 cells with 0, 10, 20, and 30 μM GL-V9 for 24 h. Scale bar: 25 μm; (**D**) Western blotting analysis of pro-caspase 3, cleaved caspase 3, pro-caspase 9, cleaved caspase 9, cleaved caspase 8, and PARP-1 after treatment of BXPC-3 and PANC-1 cells with 10, 20, and 30 μΜ GL-V9 for 24 h. ACTB was used as the loading control; (* *p* < 0.05, ** *p* < 0.01, *** *p* < 0.001); (**E**) Annexin V-FITC/PI double staining determined by flow cytometry after pre-treatment of BXPC-3 and PANC-1 cells with 10 μM Z-VAD-FMK for 2 h followed by co-treatment with 30 μM GL-V9 for 24 h; (**F**) Western blot analysis of Bcl-2, Mcl-1, Bcl-xL, and Bax was performed after treating BXPC-3 and PANC-1 cells with 0, 10, 20, or 30 μM GL-V9 for 24 h. ACTB was used as the loading control (ns: no significance, * *p* < 0.05, ** *p* < 0.01, *** *p* < 0.001); (**G**) JC-1 staining was assessed using flow cytometry after treatment of BXPC-3 and PANC-1 cells with 0, 10, 20, and 30 μM GL-V9 for 24 h. Quantitative analysis of JC-1 fluorescence (red fluorescence or green fluorescence) was performed (mean ± SD. ** *p* < 0.01); (**H**) quantitative analysis of ATP production was conducted following treatment of BXPC-3 and PANC-1 cells with 0, 10, 20, and 30 μΜ GL-V9 for 24 h. Data represent mean ± SD. ** *p* < 0.01; (**I**) the ROS level was analyzed by flow cytometry using DCFH-DA probe after treatment of BXPC-3 and PANC-1 cells with 0, 10, 20, and 30 μM GL-V9 for 24 h. Quantitative analysis was conducted, (mean ± SD. ** *p* < 0.01); (**J**) Western blot analysis of Cyt C in mitochondria and cytosol after BXPC-3 and PANC-1 cells were treated with GL-V9 (0, 10, 20, or 30 μM) for 24 h. VDAC1 was used as mitochondria control. β-Tubblin was used as cytosol control (ns: no significance, ** *p* < 0.01, *** *p* < 0.001).

In our previous study, we developed GL-V9, a synthetic flavonoid compound derived from wogonin. It effectively inhibited the growth of various tumor cells and induced their apoptosis. However, the effect of GL-V9 in PDAC cells has not yet been investigated. As shown in Figure 1A, GL-V9 exhibited a greater ability to suppress the proliferation of BXPC-3 and PANC-1 cells. The IC_50_ values for 24 h treatment with GL-V9 on BXPC-3 and PANC-1 cells were 15.49 ± 0.73 μM and 16.86 ± 1.17 μM, respectively. Additionally, the IC_50_ values of 48 h GL-V9 treatment for BXPC-3 and PANC-1 cells were 6.96 ± 1.33 μM and 9.83 ± 2.07 μM, respectively. Based on the IC50 values observed after 24 h of exposure, the doses selected for subsequent studies were determined to be 10 μM, 20 μM, and 30 μM. To investigate the apoptosis-inducing effect of GL-V9, AnnexinV-FITC/PI double staining and DAPI staining were performed. The rates of apoptosis (including early and late phases) are shown in Figure 1B. GL-V9 induced apoptosis in both BXPC-3 and PANC-1 cells in a concentration-dependent manner. After treatment with 30 μM GL-V9 for 24 h, BXPC-3 and PANC-1 cells had bright blue, fluorescent spots (Figure 1C), indicating nuclear condensation, chromatin agglutination, and the presence of apoptotic bodies in the late apoptotic stage.

Our prior studies demonstrated that GL-V9 has the potential to initiate mitochondrial apoptosis in tumor cells. Consequently, we measured the levels of caspase family proteins in BXPC-3 and PANC-1 cells. After a 24 h treatment with GL-V9, a dose-dependent decrease in pro-caspase 9, pro-caspase 3, and PARP-1 levels was observed (Figure 1D). Conversely, those of cleaved caspase 9, cleaved caspase 3, and PARP-1 were increased in their active forms. Z-VAD-FMK, a broad-spectrum caspase inhibitor, partially reversed GL-V9-induced apoptosis, suggesting that GL-V9 induces caspase 9-dependent apoptosis (Figure 1E). The levels of anti-apoptotic proteins, including Bcl-2, Mcl-1, and Bcl-xL, decreased in a dose-dependent manner, while the level of the pro-apoptotic protein Bax increased in a dose-dependent manner (Figure 1F). GL-V9 induced the loss of MMP (Figure 1G), reduced ATP levels in a dose-dependent manner (Figure 1H), and increased ROS levels in the two PDAC cell lines (Figure 1I). Following treatment with GL-V9 (30 μM), Cyt c levels in the mitochondria decreased, whereas those in the cytosol increased (Figure 1J). In summary, GL-V9 can induce mitochondrial apoptosis in PDAC cells, resulting in mitochondrial dysfunction and oxidative stress.

### 2.2. GL-V9 Induces Apoptosis via Autophagy-Mediated Degradation of YAP1 in PDAC Cells

**Figure 2:**
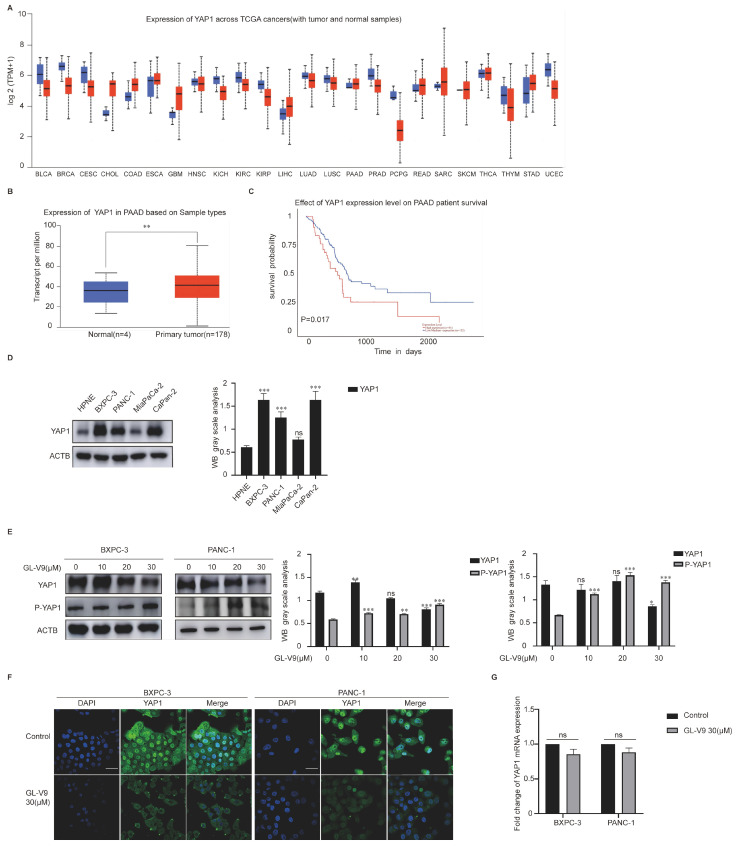
GL-V9 induces apoptosis through decreasing the expression of YAP1 in PDAC cells. (**A**) Analysis of the TCGA database revealed significant variation in YAP1 expression across different cancer types; (**B**) the expression of YAP1 in pancreatic cancer clinical samples based on the TCGA database (** *p* < 0.01); (**C**) the effect of YAP1 expression on pancreatic cancer patients’ survival; (**D**) Western blot analysis of YAP1 expression in HPNE, BXPC-3, PANC-1, MiaPaCa-2, and CaPan-2 cells (ns: no significance, *** *p* < 0.001); (**E**) Western blot analysis of YAP1 and P-YAP1 after BXPC-3 and PANC-1 cells were treated with GL-V9 (0, 10, 20, or 30 μM) for 24 h (ns: no significance, * *p* < 0.05, ** *p* < 0.01, *** *p* < 0.001); (**F**) the effect of GL-V9 on nuclear localization of YAP1 (scale bar, 25 μm); (**G**) the effect of GL-V9 on YAP1 mRNA level in pancreatic cancer cells (ns: no significance).

YAP1 acts as a transcriptional coactivator and can suppress the transcription of genes related to apoptosis. Analysis of the TCGA database revealed that the YAP1 levels are significantly higher in various tumors, including CHOL, COAD, LIHC, PAAD, READ, SARC, SKCM, and STAD (Figure 2A, the blue columns represent normal tissue, and the red columns represent tumor tissue). In our previous studies, we found that GL-V9 has a good therapeutic effect on PDAC. Therefore, we studied the effect and mechanism of GL-V9 on PDAC. Clinical samples of human pancreatic cancer showed significantly increased YAP1 levels compared to those of the normal population (Figure 2B, normal samples: n = 4, pancreatic primary tumor samples: n = 178). The high expression of YAP1 was negatively correlated with survival prognosis (Figure 2C) and was confirmed in various human pancreatic cancer cell lines (HPNE, BXPC-3, PANC-1, Capan-2, and Miapaca-2) (Figure 2D). GL-V9 decreased the expression of YAP1 and increased that of P-YAP1 in a dose-dependent manner (Figure 2E) in both BXPC-3 and PANC-1 cells but had no significant effect on YAP1 expression in Miapaca-2. The IF results demonstrated that GL-V9 significantly inhibited the nuclear translocation of YAP1 (Figure 2F). However, GL-V9 did not have any effect on the mRNA levels of YAP1 (Figure 2G). We hypothesize that GL-V9 reduces YAP1 protein levels by promoting its degradation.

**Figure 3:**
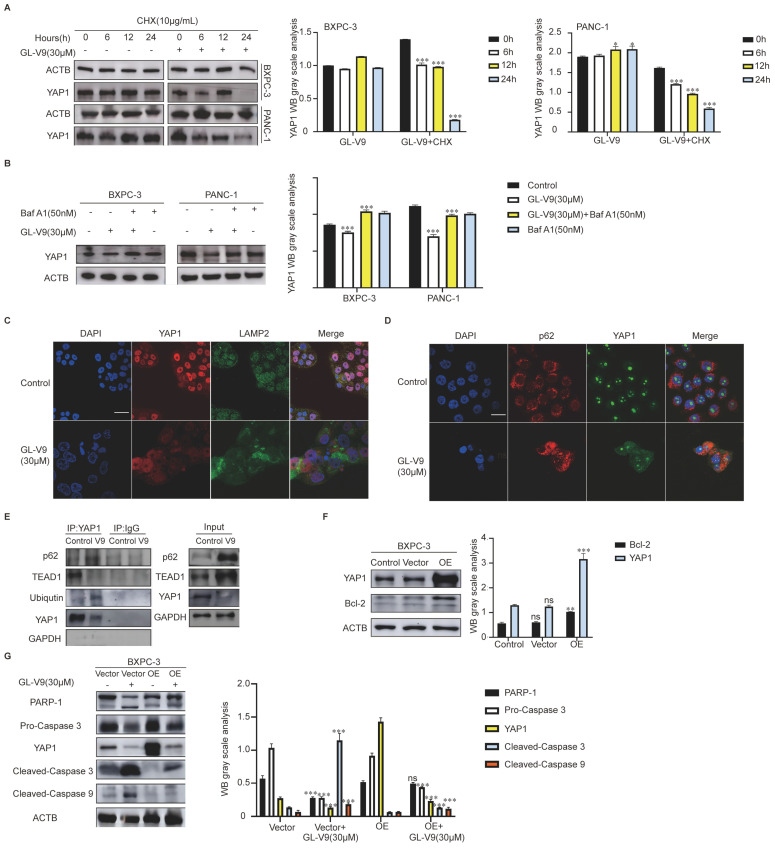
GL-V9 induces degradation of YAP1 through the autophagy–lysosome pathway. (**A**) The impact of GL-V9 on the stability of YAP1 protein was investigated. BXPC-3 and PANC-1 cells were treated with 10 μg/mL CHX and 30 μM GL-V9 for 0, 6, 12, and 24 h (* *p* < 0.05, *** *p* < 0.001); (**B**) the effect of Baf A1 on YAP1 protein by GL-V9. BXPC-3 and PANC-1 cells were pre-treated with 50 nM for 2 h and then co-treated with 30 μM GL-V9 for 24 h (*** *p* < 0.001); (**C**) the effect of GL-V9 on the location of YAP1 and LAMP2, (scale bar, 25 μm); (**D**) the effect of GL-V9 on the location of YAP1 and p62, (scale bar, 25 μm); (**E**) the effect of the binding of YAP1 with ubiquitin, p62, and TEAD1 induced by GL-V9; (**F**) the expression of YAP1 and Bcl-2 in YAP1 overexpression BXPC-3 cells (ns: no significance, ** *p* < 0.01, *** *p* < 0.001); (**G**) YAP1 overexpression reversed the level of apoptosis protein induced by GL-V9 (ns: no significance, *** *p* < 0.001).

Then we monitored the degradation level of YAP1 protein by combining cycloheximide (CHX) with GL-V9. YAP1 expression was significantly decreased after treatment with GL-V9 for 24 h (Figure 3A). After the addition of Baf A1, an autophagy inhibitor, the GL-V9-induced reduction in YAP1 protein was effectively reversed (Figure 3B). GL-V9 significantly reduces the presence of YAP1 in the nucleus and promotes its translocation into the cytoplasm. In the cytoplasm, YAP1 and LAMP2 exhibit evident colocalized yellow spots (Figure 3C). As an autophagy receptor protein, p62 connects to ubiquitinated proteins on one side and directs them to autophagosomes on the other side. This process induces the degradation of proteins through autophagy. GL-V9 significantly increased the co-localization of YAP1 and p62 in the cytoplasm (Figure 3D). These findings suggest that the GL-V9-induced decrease in YAP1 is attributed to autophagy–lysosome pathway-mediated degradation. What is more, after GL-V9 treatment, the interaction between YAP1 and TEAD1 was diminished, and the association of YAP1 with Ubiquitin and p62 was enhanced (Figure 3E). The overexpression of YAP1 in BXPC-3 cells results in the upregulation of Bcl-2 (Figure 3F) and has the potential to reverse the GL-V9-induced caspase cascade (Figure 3G). These results suggested that GL-V9 can degrade the YAP1 through the autophagy–lysosome pathway, thereby inducing apoptosis in PDAC cells.

### 2.3. GL-V9 Induces Autophagosome–Lysosome Fusion and Lysosomal Repair in PDAC Cells

**Figure 4:**
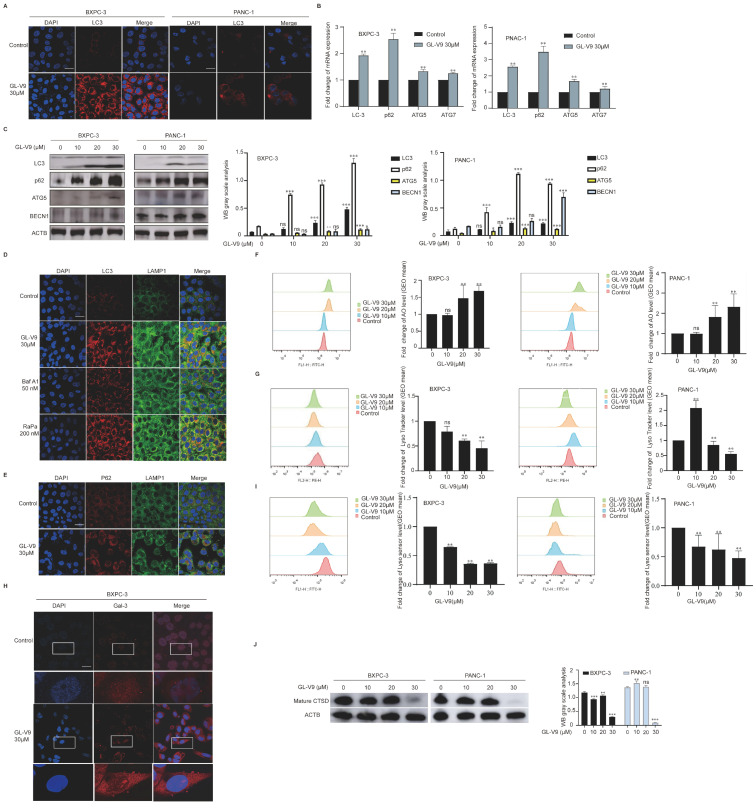
GL-V9 induces autophagosome–lysosome fusion and lysosomal repair in PDAC cells. (**A**) The effect of GL-V9 on the expression of LC3, (scale bar, 25 μm); (**B**) the effects of GL-V9 on autophagy-related genes in PDAC cells (** *p* < 0.01); (**C**) the effect of GL-V9 on the expression level of autophagy-related proteins (ns: no significance, * *p* < 0.05, ** *p* < 0.01, *** *p* < 0.001); (**D**) the content and location of LC3 and LAMP1 was determined by immunofluorescence after BXPC-3 cells were treated with GL-V9 30 μM, Baf A1 50 nM, and Rapa 200 nM for 24 h, (scale bar, 25 μm); (**E**) the content and location of p62 and LAMP1 was determined by immunofluorescence after BXPC-3 cells were treated with GL-V9 30 μM for 24 h, (scale bar, 25 μm); (**F**) the effect of GL-V9 on lysosomal membrane permeability (data represent mean ± SD. ns: no significance, ** *p* < 0.01); (**G**) the effect of GL-V9 on lysosome volume and integrity (data represent mean ± SD. ns: no significance, ** *p* < 0.01); (**H**) the effect of GL-V9 on the location of Gal-3, (scale bar, 25 μm); (**I**) the effect of GL-V9 on lysosome acidity and alkalinity (data represent mean ± SD. ** *p* < 0.01); (**J**) the effect of GL-V9 on the expression of Cathepsin D (ns: no significance, ** *p* < 0.01, *** *p* < 0.001).

To investigate the effect of GL-V9 on autophagy in PDAC cells, we performed immunofluorescence analysis to measure LC3 expression. After treatment with 30 μM GL-V9 for 24 h, a significant increase in the number of red fluorescence-labeled LC3 spots was observed in both BXPC-3 and PANC-1 cells (Figure 4A). GL-V9 also elevated the mRNA levels of autophagy initiation-related genes (LC3, ATG5, ATG7, and p62) and boosted the levels of autophagy-related proteins (LC3 II/I, p62, ATG5, and BECN1) (Figure 4B,C). These findings indicate that GL-V9 promotes autophagy initiation and induces autophagosome accumulation in PDAC cells. Then we utilized LC3 (red) and LAMP1 (green) to identify autophagosomes and lysosomes, respectively, and observe their fusion. As shown in Figure 4D,E, after treatment with 30 μM GL-V9, there was a significant increase in the co-localization of yellow spots between LC3 and LAMP1 and between p62 and LAMP1. These findings imply that GL-V9 could promote the fusion of autophagosomes and lysosomes in BXPC-3 cells.

Because GL-V9-induced autophagy is accompanied by an increase in the levels of p62, we hypothesized that the lysosomal-mediated autophagy degradation stage may be affected. AO staining results revealed that GL-V9 significantly increased the green fluorescence of AO in both BXPC-3 and PANC-1 cells (Figure 4F), suggesting enhanced permeability of the lysosomal membrane. GL-V9 significantly reduced the red fluorescence of Lyso Tracker Red in PDAC cells, indicating the compromised integrity of lysosomes (Figure 4G). After treatment with 30 μM of GL-V9 for 24 h, there was a noticeable change in the distribution of Gal-3 within the cytoplasm, transitioning from a dispersed pattern to a blotch-like accumulation (Figure 4H). This suggests that GL-V9 could induce damage to lysosomal membranes, subsequently facilitating the repair of the Gal-3 membrane. Moreover, GL-V9 induced lysosomal alkalization and reduced the levels of mature CTSD in a dose-dependent manner (Figure 4I,J). These findings suggest that GL-V9 may induce lysosomal dysfunction in PDAC cells, resulting in the induction of autophagy and repair of damaged lysosomes.

### 2.4. GL-V9 Induces Mitochondrial Apoptosis by Promoting AKT/mTOR/TFEB-Mediated Autophagy

**Figure 5:**
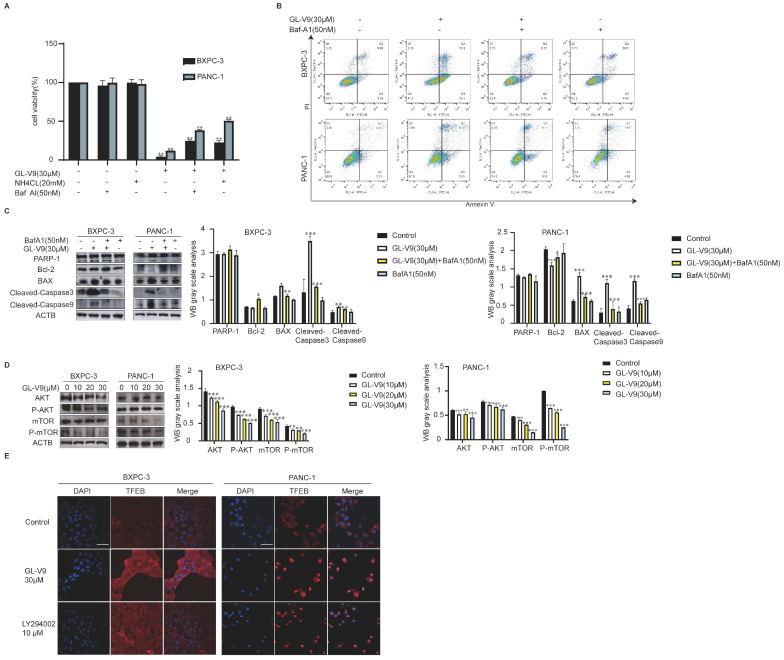
GL-V9 induces mitochondrial apoptosis by promoting AKT/mTOR/TFEB-mediated autophagy. (**A**) The impact of the autophagy inhibitor in conjunction with GL-V9 on the proliferation of pancreatic cancer cells was evaluated using the MTT assay. BXPC-3 and PANC-1 cells were exposed to 20 mM NH4Cl and 50 nM Baf A1, and were co-treated with 30 μM GL-V9 for 24 h (mean ± SD. ** *p* < 0.01); (**B**) flow cytometry was used to assess Annexin V-FITC/PI double staining following a 2 h pre-treatment of BXPC-3 and PANC-1 cells with 50 nM Baf A1, followed by co-treatment with 30 μM GL-V9 for 24 h; (**C**) Western blot analysis of PARP-1, Bcl-2, cleaved caspase 3, Bax, and cleaved caspase 9 was performed after pre-treating BXPC-3 and PANC-1 cells with 50 nM Baf A1 for 2 h and then co-treating them with 30 μM GL-V9 for a duration of 24 h. ACTB was used as a loading control (* *p* < 0.05,** *p* < 0.01, *** *p* < 0.001); (**D**) Western blot analysis of AKT, P-AKT, mTOR, and P-mTOR after BXPC-3 and PANC-1 cells were treated with GL-V9 (0, 10, 20, and 30 μM) for 24 h. ACTB was used as loading control (** *p* < 0.01, *** *p* < 0.001); (**E**) the content and location of TFEB was determined by immunofluorescence after BXPC-3 and PANC-1 cells were treated with GL-V9 30 μM. Scale bar, 25 μm.

The relationship between autophagy and apoptosis induced by GL-V9 in PDAC cells was further investigated using autophagy inhibitors Baf A1 and NH4Cl. After treatment with Baf A1 and NH 4Cl, the growth inhibition induced by GL-V9 in PDAC cells was significantly reversed (Figure 5A). AnnexinV-FITC/PI double-staining results showed that Baf A1 significantly reversed the apoptosis induced by GL-V9 (Figure 5B). The upregulation of Bax and downregulation of Bcl-2 by GL-V9 was effectively reversed by Baf A1, and the cascade reaction of caspase was reversed (Figure 5C). Our previous study demonstrated that GL-V9 could regulate various functions through the AKT signaling pathway in different types of tumor cells [22]. Thus, we hypothesized that GL-V9 may regulate autophagy in human pancreatic cancer cells through the AKT/mTOR pathway. The expression of P-AKT, AKT, P-mTOR, and mTOR showed a dose-dependent decrease (Figure 5D), suggesting that GL-V9 effectively suppressed the AKT/mTOR pathway in both BXPC-3 and PANC-1 cells. TFEB is a key transcription factor in regulating autophagy, and its activity is inhibited by the mTORC1 complex. GL-V9 facilitated the translocation of TFEB from the cytoplasm to the nucleus (Figure 5E), thereby enhancing its role in promoting autophagy. These findings suggest that GL-V9 promotes autophagy through the AKT/mTOR/TFEB pathway, ultimately leading to mitochondrial apoptosis in PDAC cells.

### 2.5. GL-V9 Inhibits PDAC Growth In Vivo by Activating Autophagy and Inducing Apoptosis

**Figure 6:**
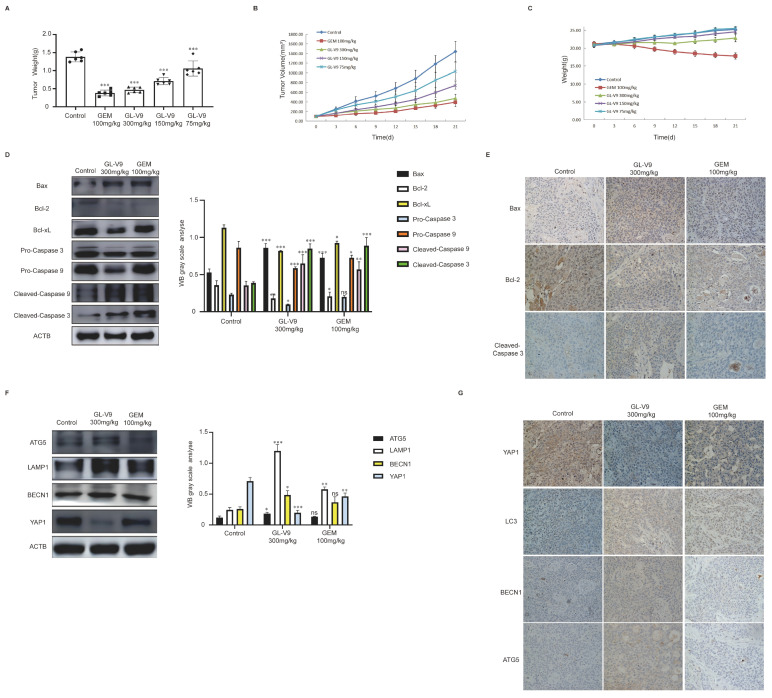
GL-V9 inhibits the growth of PDAC in vivo via activating autophagy and inducing apoptosis. (**A**) The weight of tumors for six groups of animals were compared. Data represent mean ± SD. *** *p* < 0.001; (**B**) the tumor volume was measured once every three days; (**C**) the body weight of mice was measured once every three days within 27 days of administration; (**D**) Western blot analysis of Bax, Bcl-2, Bcl-xL, pro-caspase 3, pro-caspase 9, cleaved caspase 9, and cleaved caspase 3 in tumor xenograft tissues. ACTB was used as loading control (ns: no significance, * *p* < 0.05, ** *p* < 0.01, *** *p* < 0.001); (**E**) the expression of apoptosis-related proteins in tumor xenograft tissues were measured by immunohistochemistry. Scale bars, 50 μm; (**F**) Western blot analysis of ATG5, LAMP1, BECN1, and YAP1 in tumor xenograft tissues. ACTB was used as loading control (ns: no significance, * *p* < 0.05, ** *p* < 0.01, *** *p* < 0.001); (**G**) the expression of autophagy-related proteins in tumor xenograft tissues was measured by immunohistochemistry. Scale bars, 50 μm.

To validate the findings of in vitro studies, we further investigated the anticancer effects of GL-V9 on tumor xenograft models using human BXPC-3 cell lines. GL-V9 significantly inhibited the growth of tumors in nude mice, and treatment with 300 mg/kg GL-V9 was as effective as the positive drug GEM (100 mg/kg) (Figure 6A,B). GL-V9 treatments had negligible influence on the body weight of the animals (Figure 6C). To investigate the anti-cancer mechanism of GL-V9 against pancreatic tumor growth in vivo, we measured the levels of apoptosis- and autophagy-associated proteins using WB and IHC. GL-V9 significantly increased the levels of cleaved caspase 3, cleaved caspase 9, and Bax (Figure 6D) and significantly decreased those of Bcl-2, Bcl-xL, pro-caspase 3, and pro-caspase 9. IHC results showed an increase in the levels of Bax and cleaved caspase 3 and a decrease in that of Bcl-2 (Figure 6E). Moreover, GL-V9 significantly increased the expression of ATG5, LAMP1, and BECN1. In vitro results confirmed that YAP1 is a substrate for GL-V9-induced autophagy degradation, resulting in a significant decrease in its protein levels. This finding was successfully validated in vivo, confirming that GL-V9 treatment reduced the protein levels of YAP1 (Figure 6F,G).

## 3. Discussion

PDAC is the most malignant gastrointestinal tumor and has a poor prognosis. Previously, we found that GL-V9 effectively fights solid tumors, such as breast cancer, liver cancer, colorectal cancer, and T-lymphoblastoma [21,22,25,26]. However, the therapeutic effect of GL-V9 on PDAC has not yet been studied. Therefore, this paper investigates the effect and mechanism of GL-V9 on PDAC through in vivo and in vitro experiments, providing strong evidence for its expanded therapeutic use.

Our latest study showed that GL-V9 promotes YAP1 degradation through the autophagy–lysosome pathway and induce mitochondrial apoptosis in PDAC cells. The mitochondria play a crucial role in apoptosis [27,28]. Our earlier research has shown that GL-V9 is more effective than wogonin in suppressing growth and inducing mitochondria-mediated apoptosis in human breast cancer and hepatocellular carcinoma cells [24,25]. We found that GL-V9 induces dose-dependent apoptosis in human PDAC cells. It increases ROS levels, decreases MMP, causes Cyt C translocation, and triggers a caspase 9-mediated cascade that activates mitochondrial apoptosis.

YAP/TAZ is a key protein in the Hippo pathway, which drives the progression and metastasis of PDAC [29,30]. Recent studies have showed that activating autophagy can effectively block the Hippo pathway [31,32]. Analysis of human clinical samples from the TCGA database shows that YAP1 is overexpressed in pancreatic cancer patients, and higher YAP1 expression is associated with lower overall survival rates. In this study, we confirmed the in vitro overexpression of YAP1 in human PDAC cells. YAP1 is a context-specific driver for PDAC [33], and its activation allows pancreatic cancer cells to bypass oncogenic KRAS dependency [34]. GL-V9 reduced the expression and nuclear localization of YAP1, as well as its binding to TEAD1. Overexpression of YAP1 resulted in an increased expression of Bcl-2 and significantly reversed GL-V9-induced apoptosis. GL-V9 treatment did not significantly affect YAP1 mRNA levels, indicating no impact on YAP1 synthesis. Protein stability tests showed an increased degradation rate of YAP1 in the GL-V9 treatment group. Regarding the relationship between autophagy and YAP1 [35,36], GL-V9 increased the co-localization of YAP1 with LAMP2 and p62, and the autophagy inhibitor BafA1 reversed the GL-V9-induced decrease in YAP1. These findings suggest that GL-V9 induces apoptosis in PDAC cells through the autophagy-mediated degradation of YAP1.

PDAC is characterized by autophagy-dependent tumorigenic growth [37]. Given the complex interaction and regulation between autophagy and apoptosis [38], we evaluated the effects of GL-V9 on autophagic flux. GL-V9 enhanced the accumulation of autophagosomes, facilitated their fusion with lysosomes, disrupted lysosomal functionality, and inhibited the degradation phase of autophagy. Further investigation revealed that GL-V9-induced apoptosis in human PDAC cells could be reversed by autophagy inhibitors BafA1 or NH4Cl, suggesting that GL-V9 induces apoptosis by promoting autophagy. The initiation and execution of autophagy is regulated by a variety of signaling pathways, one of which is the AKT/mTOR pathway [39]. By detecting the protein levels related to the AKT/mTOR pathway, we found that GL-V9 significantly reduced the expression of AKT, p-AKT, mTOR, and p-mTOR. TFEB is a transcription factor that controls the expression of autophagy-related genes [40]. GL-V9 treatment causes TFEB to move from the cytoplasm to the nucleus, promoting lysosome regeneration and its biological function. These findings suggest that GL-V9 has the potential to enhance autophagy in human PDAC cells by modulating the AKT/mTOR/TFEB pathway.

Gemcitabine, a first-line treatment for PDAC [41], is often associated with severe side effects and drug resistance [42,43], which greatly affect the prognosis of patients, thus limiting the dose and cycle of chemotherapy. The in vivo results indicate that GL-V9 effectively inhibits tumor growth in BXPC-3 cell-transplanted nude mice, with its highest dose showing similar efficacy to gemcitabine and low toxicity. Additionally, levels of cleaved caspase 3, cleaved caspase 9, Bax, and autophagy-related proteins LC3, ATG5, and BECN1 were up-regulated, while Bcl-2 and YAP1expression decreased. However, the in vivo experimental results only provided preliminary evidence of autophagy and apoptosis in this tumor model, and the specific mechanism by which GL-V9 reduces YAP1 expression still needs further verification.

## 4. Materials and Methods

### 4.1. Cell Lines and Cell Culture

BXPC-3 and PANC-1, MiaPaCa-2, Capan-2, HPNE, and 293T cells were obtained from the Shanghai Institute of Biochemistry and Cell Biology, cultured in RPMI-1640 (GIBCO, Stockton, CA, USA) and DMEM (GIBCO, CA, USA) supplemented with 10% FBS, maintained at 37 °C, 5% CO_2_. Passaging was performed using a mixture of EDTA buffer and 0.25% trypsin.

### 4.2. Reagents

GL-V9 (C_24_H_27_NO_5_, 98% purity), synthesized by Prof. Zhiyu Li in our lab, was used in this study. For in vitro experiments, GL-V9 was dissolved in DMSO to a concentration of 0.02 M, stored at −80 °C, and diluted with RPMI-1640 medium. For in vivo experiments, GL-V9 was formulated for intragastric administration using sodium carboxymethyl cellulose (CMC-Na) at a concentration of 300 mg/kg. Gemcitabine (GEM) was purchased from Hansoh Pharma Co., Ltd. (Lianyungang, China) (LOT: 619201121).

### 4.3. PDAC Graft Tumor Model

BALB/c nude mice (female, 6–8 weeks old) were purchased from Changzhou Cavens Laboratory Animal Co., Ltd. (Changzhou, China) (No. SCXK (SU)-2016-0010), at a weight of 20 ± 2 g. All mice were acclimated to a standard rearing environment (temperature: 25 ± 1 °C, humidity: 40–60%, keeping 12 h of light.) for 1 week before experiments were carried out. This animal study received approval from the Ethics Committee of China Pharmaceutical University. The ethical approval number is 202209A037, and the date of approval is 21 September 2022.

A total of 1 × 10^6^ BXPC-3 tumor cells were suspended in 0.1 mL of serum-free medium and injected subcutaneously into the flanks of the mice. The tumor-bearing nude mice were then randomly split into 5 groups according to their weight (n ≥ 6): (1) control; (2) GL-V9 (300 mg/kg/day), (3) GL-V9 (150 mg/kg/day); (4) GL-V9 (75 mg/kg/day); (5) GEM (100 mg/kg/7 days). GL-V9 (75 mg/kg/day, 150 mg/kg/day, or 300 mg/kg/day) was administered orally and gemcitabine (100 mg/kg/7 days) was administered intraperitoneally for twenty-one days. The control group received 0.9% normal saline. The volume of intragastric administration was 0.1 mL/10 g to each mouse. The mice were sacrificed, and the tumor tissue was cleaned, fixed, and embedded in paraffin for sectioning.

### 4.4. Western Blot

Western blotting and immunoprecipitation assays were conducted as previously described [44]. The primary antibodies used in Western blotting included ACTB, Bax, Bcl-2, Bcl-xL, caspase 3, caspase 8, caspase 9, Cyt C, GAPDH, Mcl-1, mTOR, PARP-1, P-YAP1, VDAC1, YAP1 and β-tubblin (1:1000, ABclonal, Wuhan, China), ATG5 and BECN1 (1:1000, Bioswap, Hangzhou, China), TEAD1 (1:1000, FineTest, Wuhan, China), Ubiquitin (1:1000, Immunoway, Plano, TX, USA) AKT, Gal-3, LAMP1, LAMP2, LC3, p62, P-AKT, and TFEB (1:1000, Proteintech, Wuhan, China). Each blot shows one representative out of at least three.

### 4.5. Annexin V/PI Staining Assay

BXPC-3 and PANC-1 cells were collected, stained using the Annexin V/PI Cell Apoptosis Detection Kit (Vazyme Biotech Co., Ltd., Nanjing, China), and analyzed with AccuriTMC6 flow cytometry and CellQuest software (version 5.1). Cells that showed no positive staining for Annexin V or PI were deemed viable.

### 4.6. Mitochondrial Isolation and Protein Extraction

The Mitochondrial Isolation and Protein Extraction Kit (Proteintech Group, Inc., Wuhan, China) was used to extract mitochondria according to manufacturer’s instructions [45]. Data acquisition was conducted using AccuriTMC6 flow cytometry and CellQuest software.

### 4.7. ROS Measurement

BXPC-3 and PANC-1 cells were collected and stained using the ROS Assay Kit (Beyotime Biotechnology, Shanghai, China). The cells were subsequently incubated in the dark at 37 °C for 20 min with DCFH-DA dye, which was previously diluted to 1:1000 in serum-free DMEM.

### 4.8. Detection of the Loss of Mitochondrial Membrane Potential

Alterations in mitochondrial membrane potential (MMP), a key indicator of apoptosis, were measured using the JC-1 MMP Assay Kit (Beyotime Biotechnology, Shanghai, China). A total of 3 × 10^5^ human pancreatic cancer cells were inoculated into a 6-well plate, and after the cells were attached to the wall, the appropriate drugs were added. A total of 24 h later, the supernatant was collected, washed with cold PBS twice, and digested with EDTA trypsin. The cells were collected by centrifugation, the supernatant was discarded, and the cells were re-suspended with a prepared JC-1 probe and incubated in a metal bath at 37 °C for 20 min, avoiding light, and mixing up and down every 10 min. After incubation, the cells were washed with JC-1 staining buffer. Subsequently, the cells were resuspended in 300 μL of PBS and then subjected to screening and analysis using the flow cytometry instrument.

### 4.9. ATP Assay

Intracellular ATP levels were assessed using the ATP Detection Assay Kit (Beyotime Biotechnology, Shanghai, China). A total of 200 μL of cell lysate was added to BXPC-3 and PANC-1 cells, lysed on ice for 5 min, centrifuged at 12,000× *g* for 5 min, and 20 μL of supernatant was taken to measure BCA and leveling protein concentration with cell lysate. A total of 100 μL of ATP assay working solution (dilute the probe 1:100) was added and left at room temperature for 5 min; then 20 μL of the sample to be tested was added and the absorbance was detected on Varioskan Flash Full Wavelength Enzyme Labeler (Thermo, Waltham, MA, USA).

### 4.10. Real-Time PCR Analysis

The extracted RNA was reverse transcribed into cDNA following the instructions provided by the reverse transcription kit (Vazyme Biotech Co., Ltd., Nanjing, China), the reaction was prepared according to the instructions of the SYBR Green I reagent, and the PCR reaction was carried out using Applied Biosystems QuanStudio3. The primer sets were as follows:**Name****Sequence**LC3 forwardAACATGAGCGAGTTGGTCAAGLC3 reverseGCTCGTAGATGTCCGCGATp62 forwardGCACCCCAATGTGATCTGCp62 reverseCGCTACACAAGTCGTAGTCTGGATG5 forwardAAAGATGTGCTTCGAGATGTGTATG5 reverseCACTTTGTCAGTTACCAACGTCAATG7 forwardATGATCCCTGTAACTTAGCCCAATG7 reverseCACGGAAGCAAACAACTTCAACGAPDH forwardCGGAGTCAACGGATTTGGTCGTATGAPDH reverseAGCCTTCTCCATGGTGGTGAAGACYAP1 forwardGGATTTCTGCCTTCCCTGAAYAP1 reverseGATAGCAGGGCGTGAGGAAC

### 4.11. Immunofluorescence (IF)

The cells were fixed with 4% paraformaldehyde for 10 min, permeabilized using 0.3% Triton for another 10 min, and subsequently blocked with 3% BSA for 2 h at room temperature. They were incubated overnight at 4 °C with a 1:100 dilution of primary antibodies followed by a 1:100 dilution of fluorescent secondary antibodies. The mixture was dark incubated at room temperature for 40 min before being treated with DAPI anti-quencher (Beyotime Biotechnology, China). Finally, the cells were examined with a laser scanning confocal microscope (Fluoview FV1000, Olympus, Tokyo, Japan).

### 4.12. Tissue Staining

Lysosomal membrane permeability alterations were analyzed by acridine orange localization fluorescence in lysosomes or cytosol. For acridine orange (AO) staining, the lysosomes were incubated with 5 μM of AO in a metal bath at 37 °C for 20 min, washed with PBS, and assayed on the machine. Lysotracker Red and LysoSensor Green enable the localization of lysosomes and the detection of intracellular lysosomal pH. The fluorescent probe was incubated with 1 μM Lyso Tracker Red in a metal bath at 37 °C for 20 min, followed by two washes with PBS for 5 min each, and screened for on-line assay; the same conditions were used for LysoSensor Green.

### 4.13. Co-Immunoprecipitation

BXPC-3 cells were treated with 0 and 30 μM of GL-V9 for 24 h. A Co-IP toolkit (P2179S, beyotime, Shanghai, China) was used to evaluate protein–protein interaction. BXPC-3 cells were lysed on ice and centrifuged at 13,000× *g* and 4 °C for 10 min. The resulting supernatant was collected and incubated with the bait antibody (anti-YAP1, 1–2 μg per 100–500 μg of total protein, Abclonal) or IgG on a vertical rotating mixer at 4 °C overnight. Protein A/G magnetic beads were added and co-incubated at 4 °C for 3 h. The supernatant was then collected for Western blot.

### 4.14. Immunohistochemistry

The proteins expression in tumor tissues was assessed using the PV-9000 kit (Zhong Shan-Golden Bridge Biological Technology, Beijing, China). Tumor tissues were sectioned to 4 µm, deparaffinized with xylene, and rehydrated with alcohol. The sections were then treated using the streptavidin peroxidase-conjugated method for protein visualization, followed by incubation with specific primary antibodies targeting the proteins of interest. The primary antibodies used were as follows: Bax, Bcl-2, cleaved caspase 3, and YAP1 (1:1000, ABclonal); ATG5 and BECN1 (1:1000, Bioswap); and LC3 (1:1000, Proteintech).

### 4.15. Tissue Protein Extraction

Take a 50 mg tumor sample and add 200 μL of RIPA lysate, which contains protease and phosphatase inhibitors. Grind the mixture in a pre-sterilized and pre-cooled homogenizer until the tissue is completely broken up without lumps. Transfer the homogenized sample into a 1.5 mL tube and lyse it on ice for 30 min, vortexing every 10 min at 15,000 rpm. Centrifuge the mixture at 4 °C for 20 min, collect the supernatant, and determine the protein concentration using the BCA method. Then, add 5× Loading Buffer, denature the sample at 100 °C for 10 min, allow it to cool to room temperature on ice, and store at −20 °C.

### 4.16. Construction of Stable Transient Cells

Experiments were performed according to the lentivirus packaging kit (Yeasen Biotechnology, Shanghai, China). 293T cells in the logarithmic growth phase were added into six-well plates and packaged when the cells were attached to the wall and the density had reached 80%. Fresh medium was changed 2 h in advance. In a preparation system of 200 μL, 2 μL of viral plasmid, 2 ug of target plasmid, and 60 μL of Hg Transgene Transfection Reagent were mixed and left to stand for 20 min. Then, it was added dropwise in a circle in a six-well plate. The fluorescence intensity was observed after 24 h, and the virus supernatant was collected by centrifugation at 48 h. The virus supernatant was filtered by a 0.22 μm filter tip and stored at −80 °C. Lentiviral infected target cells were added with a ratio of virus supernatant: complete medium =1:1. Polybrene at a final concentration of 10 μM was added to the system, and finally, 4 ug/mL puromycin was used to screen stable transfected cells.

### 4.17. Statistical Analysis

The data were expressed as the mean ± SD. The differences between groups were assessed by a one-way analysis of variance (ANOVA) using IBM SPSS Statistics 18.0 software. *p* < 0.05 was considered statistically significant, and *p* < 0.01 was considered highly significant. GraphPad Prism 8.0.2 software was used to construct graphs.

## 5. Conclusions

Our study is the first to report that GL-V9 can induce mitochondrial apoptosis in BXPC-3 and PANC-1 cells. Mechanistic studies have shown that GL-V9 promotes autophagy through the AKT/mTOR/TFEB pathway. This autophagy induces YAP1 degradation, subsequently leading to apoptosis. The in vivo model confirmed that GL-V9 has a superior anti-cancer effect on the BXPC-3 nude mouse xenograft model and demonstrates high safety. These findings indicate that GL-V9 is a strong candidate for PDAC treatment and could be used alongside chemotherapy as an additional treatment. However, although this paper has revealed that GL-V9 can induce autophagy, the specific mechanism linking lysosomal dysfunction and autophagy induction remains to be further explored, particularly in the context of detecting the autophagy stage.

## Data Availability

The data sets generated and/or analyzed during the current study are available from the corresponding author upon reasonable request.

## Abbreviations

PDAC, pancreatic ductal adenocarcinoma; ROS, reactive oxygen species; CHX, cycloheximide; DMSO, dimethyl sulfoxide; CMC-Na, sodium carboxymethyl cellulose; GEM, gemcitabine; TV, tumor volume; MMP, mitochondrial membrane potential; AO, acridine orange; ATP: Adenosine Triphosphate; Baf A1: Bafilomycin A1; BSA: Boving Serum Albumin; Cyt C: cytochrome; CTSD: Cathepsin D; Cyt C: cytochrome; TFEB: Transcription factor EB; YAP1: Yes-associated Protein 1.

## References

[1] Del Chiaro M., Sugawara T., Karam S.D., Messersmith W.A. (2023). Advances in the management of pancreatic cancer. BMJ.

[2] Halbrook C.J., Lyssiotis C.A., Pasca di Magliano M., Maitra A. (2023). Pancreatic cancer: Advances and challenges. Cell.

[3] Park W., Chawla A., O’Reilly E.M. (2021). Pancreatic Cancer: A Review. JAMA.

[4] Hamanaka R.B., Chandel N.S. (2010). Mitochondrial reactive oxygen species regulate cellular signaling and dictate biological outcomes. Trends Biochem. Sci..

[5] Tischner D., Manzl C., Soratroi C., Villunger A., Krumschnabel G. (2012). Necrosis-like death can engage multiple pro-apoptotic Bcl-2 protein family members. Apoptosis Int. J. Program. Cell Death.

[6] Chen Y., McMillan-Ward E., Kong J., Israels S.J., Gibson S.B. (2008). Oxidative stress induces autophagic cell death independent of apoptosis in transformed and cancer cells. Cell Death Differ..

[7] Aman Y., Schmauck-Medina T., Hansen M., Morimoto R.I., Simon A.K., Bjedov I., Palikaras K., Simonsen A., Johansen T., Tavernarakis N. (2021). Autophagy in healthy aging and disease. Nat. Aging.

[8] Jain V., Singh M.P., Amaravadi R.K. (2023). Recent advances in targeting autophagy in cancer. Trends Pharmacol. Sci..

[9] Tewari D., Patni P., Bishayee A., Sah A.N., Bishayee A. (2022). Natural products targeting the PI3K-Akt-mTOR signaling pathway in cancer: A novel therapeutic strategy. Semin. Cancer Biol..

[10] Mohamed E., Kumar A., Zhang Y., Wang A.S., Chen K., Lim Y., Shai A., Taylor J.W., Clarke J., Hilz S. (2022). PI3K/AKT/mTOR signaling pathway activity in IDH-mutant diffuse glioma and clinical implications. Neuro-Oncology.

[11] Hua H., Kong Q., Zhang H., Wang J., Luo T., Jiang Y. (2019). Targeting mTOR for cancer therapy. J. Hematol. Oncol..

[12] Mossmann D., Park S., Hall M.N. (2018). mTOR signalling and cellular metabolism are mutual determinants in cancer. Nat. Rev. Cancer.

[13] Zhang M., Yue H., Huang X., Wang J., Li Z., Deng X. (2022). Novel Platinum Nanoclusters Activate PI3K/AKT/mTOR Signaling Pathway-Mediated Autophagy for Cisplatin-Resistant Ovarian Cancer Therapy. ACS Appl. Mater. Interfaces.

[14] Chen X., Yuan W., Li Y., Luo J., Hou N. (2020). Role of Hippo-YAP1/TAZ pathway and its crosstalk in cardiac biology. Int. J. Biol. Sci..

[15] Zanconato F., Cordenonsi M., Piccolo S. (2016). YAP/TAZ at the Roots of Cancer. Cancer Cell.

[16] Franklin J.M., Wu Z., Guan K.L. (2023). Insights into recent findings and clinical application of YAP and TAZ in cancer. Nat. Rev. Cancer.

[17] Eibl G., Rozengurt E. (2019). KRAS, YAP, and obesity in pancreatic cancer: A signaling network with multiple loops. Semin. Cancer Biol..

[18] Morvaridi S., Dhall D., Greene M.I., Pandol S.J., Wang Q. (2015). Role of YAP and TAZ in pancreatic ductal adenocarcinoma and in stellate cells associated with cancer and chronic pancreatitis. Sci. Rep..

[19] Dey A., Varelas X., Guan K.L. (2020). Targeting the Hippo pathway in cancer, fibrosis, wound healing and regenerative medicine. Nat. Rev. Drug Discov..

[20] Liu J., Guo Y., Zhang R., Xu Y., Luo C., Wang R., Xu S., Wei L. (2023). Inhibition of TRPV4 remodels single cell polarity and suppresses the metastasis of hepatocellular carcinoma. Cell Death Dis..

[21] Guo Y., Wei L., Zhou Y., Lu N., Tang X., Li Z., Wang X. (2020). Flavonoid GL-V9 induces apoptosis and inhibits glycolysis of breast cancer via disrupting GSK-3β-modulated mitochondrial binding of HKII. Free Radic. Biol. Med..

[22] Hu P., Li H., Yu X., Liu X., Wang X., Qing Y., Wang Z., Wang H., Zhu M., Xu J. (2019). GL-V9 exerts anti-T cell malignancies effects via promoting lysosome-dependent AKT1 degradation and activating AKT1/FOXO3A/BIM axis. Free Radic. Biol. Med..

[23] Yang L., He Z., Yao J., Tan R., Zhu Y., Li Z., Guo Q., Wei L. (2018). Regulation of AMPK-related glycolipid metabolism imbalances redox homeostasis and inhibits anchorage independent growth in human breast cancer cells. Redox Biol..

[24] Li L., Lu N., Dai Q., Wei L., Zhao Q., Li Z., He Q., Dai Y., Guo Q. (2011). GL-V9, a newly synthetic flavonoid derivative, induces mitochondrial-mediated apoptosis and G2/M cell cycle arrest in human hepatocellular carcinoma HepG2 cells. Eur. J. Pharmacol..

[25] Zhang X., Kang Y., Huo T., Tao R., Wang X., Li Z., Guo Q., Zhao L. (2017). GL-V9 induced upregulation and mitochondrial localization of NAG-1 associates with ROS generation and cell death in hepatocellular carcinoma cells. Free Radic. Biol. Med..

[26] Zhao Y., Guo Q., Zhao K., Zhou Y., Li W., Pan C., Qiang L., Li Z., Lu N. (2017). Small molecule GL-V9 protects against colitis-associated colorectal cancer by limiting NLRP3 inflammasome through autophagy. Oncoimmunology.

[27] Winter J.M., Yadav T., Rutter J. (2022). Stressed to death: Mitochondrial stress responses connect respiration and apoptosis in cancer. Mol. Cell.

[28] Bock F.J., Tait S.W.G. (2020). Mitochondria as multifaceted regulators of cell death. Nat. Rev. Mol. Cell Biol..

[29] Fukunaga Y., Fukuda A., Omatsu M., Namikawa M., Sono M., Masuda T., Araki O., Nagao M., Yoshikawa T., Ogawa S. (2022). Loss of Arid1a and Pten in Pancreatic Ductal Cells Induces Intraductal Tubulopapillary Neoplasm via the YAP/TAZ Pathway. Gastroenterology.

[30] Strnadel J., Choi S., Fujimura K., Wang H., Zhang W., Wyse M., Wright T., Gross E., Peinado C., Park H.W. (2017). eIF5A-PEAK1 Signaling Regulates YAP1/TAZ Protein Expression and Pancreatic Cancer Cell Growth. Cancer Res..

[31] Pavel M., Park S.J., Frake R.A., Son S.M., Manni M.M., Bento C.F., Renna M., Ricketts T., Menzies F.M., Tanasa R. (2021). α-Catenin levels determine direction of YAP/TAZ response to autophagy perturbation. Nat. Commun..

[32] Liang N., Zhang C., Dill P., Panasyuk G., Pion D., Koka V., Gallazzini M., Olson E.N., Lam H., Henske E.P. (2014). Regulation of YAP by mTOR and autophagy reveals a therapeutic target of tuberous sclerosis complex. J. Exp. Med..

[33] Tu B., Yao J., Ferri-Borgogno S., Zhao J., Chen S., Wang Q., Yan L., Zhou X., Zhu C., Bang S. (2019). YAP1 oncogene is a context-specific driver for pancreatic ductal adenocarcinoma. JCI Insight.

[34] Kapoor A., Yao W., Ying H., Hua S., Liewen A., Wang Q., Zhong Y., Wu C.J., Sadanandam A., Hu B. (2014). Yap1 activation enables bypass of oncogenic Kras addiction in pancreatic cancer. Cell.

[35] Desideri E., Castelli S., Dorard C., Toifl S., Grazi G.L., Ciriolo M.R., Baccarini M. (2023). Impaired degradation of YAP1 and IL6ST by chaperone-mediated autophagy promotes proliferation and migration of normal and hepatocellular carcinoma cells. Autophagy.

[36] Jin L., Chen Y., Cheng D., He Z., Shi X., Du B., Xi X., Gao Y., Guo Y. (2021). YAP inhibits autophagy and promotes progression of colorectal cancer via upregulating Bcl-2 expression. Cell Death Dis..

[37] Bryant K.L., Stalnecker C.A., Zeitouni D., Klomp J.E., Peng S., Tikunov A.P., Gunda V., Pierobon M., Waters A.M., George S.D. (2019). Combination of ERK and autophagy inhibition as a treatment approach for pancreatic cancer. Nat. Med..

[38] Chen R., Zou J., Zhong X., Li J., Kang R., Tang D. (2024). HMGB1 in the interplay between autophagy and apoptosis in cancer. Cancer Lett..

[39] Choudhury D., Ghosh D., Mondal M., Singha D., Pothuraju R., Malakar P. (2024). Polyploidy and mTOR signaling: A possible molecular link. Cell Commun. Signal..

[40] Tang H., Hou H., Song L., Tian Z., Liu W., Xia T., Wang A. (2024). The role of mTORC1/TFEB axis mediated lysosomal biogenesis and autophagy impairment in fluoride neurotoxicity and the intervention effects of resveratrol. J. Hazard. Mater..

[41] Seufferlein T., Uhl W., Kornmann M., Algül H., Friess H., König A., Ghadimi M., Gallmeier E., Bartsch D.K., Lutz M.P. (2023). Perioperative or only adjuvant gemcitabine plus nab-paclitaxel for resectable pancreatic cancer (NEONAX)-a randomized phase II trial of the AIO pancreatic cancer group. Ann. Oncol. Off. J. Eur. Soc. Med. Oncol..

[42] Rohila D., Park I.H., Pham T.V., Weitz J., Hurtado de Mendoza T., Madheswaran S., Ishfaq M., Beaman C., Tapia E., Sun S. (2023). Syk Inhibition Reprograms Tumor-Associated Macrophages and Overcomes Gemcitabine-Induced Immunosuppression in Pancreatic Ductal Adenocarcinoma. Cancer Res..

[43] Zhou T., Xie Y., Hou X., Bai W., Li X., Liu Z., Man Q., Sun J., Fu D., Yan J. (2023). Irbesartan overcomes gemcitabine resistance in pancreatic cancer by suppressing stemness and iron metabolism via inhibition of the Hippo/YAP1/c-Jun axis. J. Exp. Clin. Cancer Res..

[44] Xu Y., Zhang X., Zhang R., Sun Y., Liu J., Luo C., Yang J., Fang W., Guo Q., Wei L. (2023). AFP deletion leads to anti-tumorigenic but pro-metastatic roles in liver cancers with concomitant CTNNB1 mutations. Cancer Lett..

[45] Yue M., Hu B., Li J., Chen R., Yuan Z., Xiao H., Chang H., Jiu Y., Cai K., Ding B. (2023). Coronaviral ORF6 protein mediates inter-organelle contacts and modulates host cell lipid flux for virus production. EMBO J..

